# A Systematic Review of the Effect of Cystic Fibrosis Treatments on the Nasal Potential Difference Test in Animals and Humans

**DOI:** 10.3390/diagnostics13193098

**Published:** 2023-09-29

**Authors:** Cathalijn Leenaars, Christine Häger, Frans Stafleu, Hendrik Nieraad, André Bleich

**Affiliations:** 1 Institute for Laboratory Animal Science, Hannover Medical School, 30625 Hannover, Germany; 2 Department of Animals in Science and Society—Human-Animal Relationship, Utrecht University, 3584 CM Utrecht, The Netherlands

**Keywords:** cystic fibrosis, nasal potential difference, electrophysiology, treatment, therapy, systematic review, animal model validity

## Abstract

To address unmet treatment needs in cystic fibrosis (CF), preclinical and clinical studies are warranted. Because it directly reflects the function of the Cystic Fibrosis Transmembrane conductance Regulator (CFTR), the nasal potential difference test (nPD) can not only be used as a reliable diagnostic test for CF but also to assess efficacy of experimental treatments. We performed a full comprehensive systematic review of the effect of CF treatments on the nPD compared to control conditions tested in separate groups of animal and human subjects. Our review followed a preregistered protocol. We included 34 references: 20 describing mouse studies, 12 describing human studies, and 2 describing both. We provide a comprehensive list of these studies, which assessed the effects of antibiotics, bone marrow transplant, CFTR protein, *CFTR* RNA, directly and indirectly CFTR-targeting drugs, non-viral and viral gene transfer, and other treatments. Our results support the nPD representing a reliable method for testing treatment effects in both animal models and human patients, as well as for diagnosing CF. However, we also observed the need for improved reporting to ensure reproducibility of the experiments and quantitative comparability of the results within and between species (e.g., with meta-analyses). Currently, data gaps warrant further primary studies.

## 1. Introduction

Cystic fibrosis (CF) is a debilitating disease, with an incidence ranging from 1:2800 to 1:10,000, depending on the geographical region [1]. Whereas the cause of CF is known (i.e., mutations in the *CFTR* gene resulting in functional defects in the CFTR channel) [2], and with a large degree of understanding of the pathophysiology, there still are substantial unmet treatment needs [3,4,5]. To address these needs, new preclinical and clinical studies are still warranted.

Similarities with CF phenotype can be observed in CF animal models, for example, in electrophysiological parameters [6,7,8]. One of these parameters is the nasal potential difference (nPD), which improves when the CFTR channel function is corrected [9]. By specific changes in the protocol, the reliability of the nPD as a diagnostic test has increased [10,11]. The nPD can be measured in multiple species, e.g., mice [8], rats [6], and pigs [12], and is also used as an outcome measure in clinical trials for investigational CF treatments [9].

Preceding work from our group has shown that the predictive value of preclinical animal studies for human treatments ranges from 0% to 100% [13]. A later study found relevant differences in average predictive values between medical fields [14]. Therefore, we were interested in the predictive value of animal nPD values for human cystic fibrosis treatments. We aimed to answer this question by analyzing already published data in a systematic literature review (SR) while providing a comprehensive overview of CF treatments for which the effect on the nPD has been measured.

Based on the Cochrane handbook, we previously defined an SR as a “review comprising a full search resulting in a complete literature overview, inclusion of papers following strict criteria, tabulation of extracted data, risk of bias assessment of included studies, and meaningful (qualitative or quantitative) synthesis of the data” [15]. Our SR was designed to answer multiple review questions; in this manuscript, we focused on the data relating to potential CF treatment effects. In an elsewhere-submitted manuscript, we described the data relating to comparisons between cystic fibrosis and healthy controls [16].

Of note, methods for SRs of animal studies vary from those used for SRs of human studies (see [17] for an example of an elegant systematized review of human studies on the nPD in hypoxia-induced lung injury), mostly because the goals and experimental designs of animal studies are more variable. Whereas the development of methods for SRs of animal studies started later than that of human SR methods, several groups have proven the value of preclinical SRs in improving scientific practice [18,19,20,21,22,23], thereby decreasing the numbers of animals needed in pharmaceutical development. Because of the differences in review methods between fields, we provide an extensive description of our methods, with explanations for some of the decisions we made.

## 2. Materials and Methods

Our review was preregistered on PROSPERO (CRD42021236047) on 5 March 2021 [24]. Comprehensive searches in PubMed (comprising Medline) and Embase, unrestricted for publication date or language, were performed on 23 March 2021. A partial update was performed on 26 May 2023 and is described in the discussion. This review was reported in accordance with the PRISMA (Preferred Reporting Items for Systematic Reviews and Meta-Analyses) guidelines.

The full search strings are shown in Table 1 and follow the PICO format. The population (P) consisted of animals and/or humans; the intervention (I) could be any intervention and was not part of the search, the comparison (C) could be any between-subject comparison and was not part of the search, and the outcome (O) was the nPD. As is common for SRs of animal studies, we added a separate search component for the disease: CF. The two strings for population were combined with “OR”. The results were combined with the other two strings with “AND”.

As the topic of this SR is clearly medical, we restricted our searches to the two main medical databases. This has been shown to be a sensitive approach, and it is in line with common practice in the field of laboratory animal SRs [26,27]. For SRs of animal studies with less medical topics, we strongly suggest adding at least one more database, e.g., CABabstracts to retrieve veterinary studies, or PsychInfo for SRs in the field of neuroscience. We did not search Google Scholar because it is impossible to run reproducible searches with this search engine [28]. We explicitly excluded anything other than full peer-reviewed publications, as we were interested in experimental design affecting the nPD, and descriptions of experimental design in conference abstracts are minimal. Consequently, we did not search the grey literature.

Title-abstract screening and full-text screening were performed in a blinded manner by two independent reviewers (FS and CL) using Rayyan [29]. Screening followed the predefined criteria listed in Table 2, as per protocol [24]. Reference lists of relevant reviews and included studies were manually screened by the same reviewers for further eligible studies. Discrepancies were all resolved by discussions between the reviewers.

### 2.1. Data Extraction

Data were extracted by two independent reviewers per reference in multiple distinct phases. In the first phase (FS and HN, discrepancies: FS and CL), basic data on study design and included population were extracted in Covidence [30]. These basic data allowed us to categorize the included studies into the CF versus control comparisons that were summarized in our other publication and the treatment studies that are summarized in this manuscript. In the second phase (FS and CL), risk-of-bias data were extracted for all treatment studies, also in Covidence. In the third phase (FS and CL), only the type of treatment was extracted, straight into a Microsoft Excel spreadsheet, to efficiently create an overview of the available studies without extracting too many data that could not be analyzed. Based on this third phase, we could determine the meta-analyses to be performed per protocol. In the fourth phase, (FS and CH), details on the treatment and outcomes for this planned meta-analysis (MA) were extracted in SRDR+. In all phases, discrepancies were resolved by discussion between the reviewers.

During the last phase of data extraction, standard deviations (SDs) were converted to Standard Errors of the Mean (SEMs). When values were not provided in text or tables, one of the extractors (CH) used pixel counts with digital imaging software (GIMP2.10.30), as previously described [31]; the other (FS) used an analogue ruler. When repeated post-treatment nPDs were reported, we extracted the value closest to 24 h after treatment. These data were exported from SRDR+ to Excel.

### 2.2. Risk of Bias Assessments

Risk of bias (RoB) and study quality were assessed with various tools, as per protocol [24]. In this paper, we summarize the overall RoB for all included treatment papers, following the SYRCLE and Cochrane tools [32,33]. To prevent multiple publications of the same data sets, analyses of the reporting quality data will only be presented in a separate publication on RoB and the quality of reporting in different types of animal and human studies.

### 2.3. Analysis

Data were checked and cleaned in Excel. Cleaning comprised harmonizing spelling and capitalization. For data exported from SRDR+, cleaning additionally comprised selection of the consolidated values and merging the data into a single wide data frame. We planned to perform meta-analyses comparing treatment effects between animal models and human patients, as described in our protocol [24]. Unfortunately, the amount of data required for these analyses was not available in the current literature (as described in the results section). Thus, we restricted our analyses of the included treatment studies to narrative and quantitative summaries.

All analyses were performed in R [34] via RStudio [35], using the following packages: readxl [36], dplyr [37], ggplot2 [38], and crosstable [39].

## 3. Results

### 3.1. Study Flow and Sample

**Figure 1:**
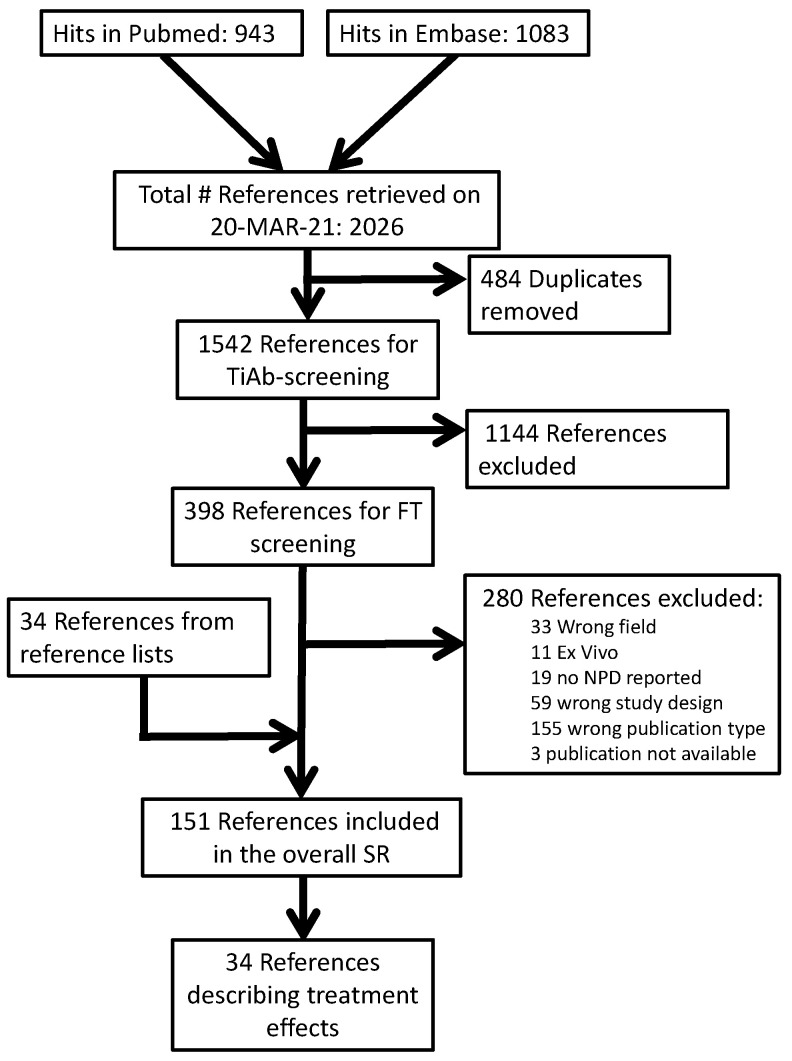
Reference flow.

Our PubMed search retrieved 943 publications, while our Embase search retrieved 1083. After the removal of 484 duplicates, 1542 titles and abstracts were screened. After excluding 1144 records in this phase, we retrieved 395 PDFs for full-text screening. Overall, 277 were excluded for the reasons shown in our overall reference flow (Figure 1). Hand searches of the reference lists of included studies and relevant reviews resulted in an additional 34 included studies.

Of the 151 references included in our overall SR, 34 described a comparison between a CF-targeting treatment and a between-subject control condition. Of these, k = 17 also described a comparison between CF and control without treatment, and these were also included in our parallel publication [16]. Full lists of the publications in each phase are available on the Open Science platform (https://doi.org/10.17605/OSF.IO/ST9MF accessed on 24 August 2023).

The here-described 34 references comprised 20 studies of CF treatments in mice, 12 of CF treatments in humans, and two studies in both species. Mouse references were published from 1993 to 2019. Human references were published from 1995 to 2014, and the two references describing both animal and human studies were from 1994 and 1996.

### 3.2. Included Treatments

**Figure 2:**
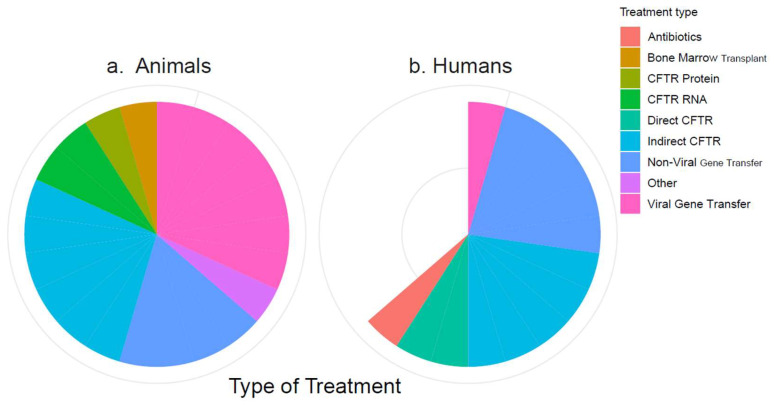
Categorization of the included treatments. (**a**) Treatments in animal studies; (**b**) treatments in human studies. The white wedge reflects the number of human studies that are “missing” compared to the number of animal studies.

The 34 included references described between one and six treatments each, which could mostly be categorized into eight types of treatment: antibiotics, bone marrow transplant, CFTR protein, *CFTR* RNA, directly CFTR-targeting drugs, indirectly CFTR-targeting drugs, non-viral gene transfer (NVGT), and viral gene transfer (VGT). Treatments that did not fit into these categories were grouped as “Other”. The numbers of included studies on these treatment types are visualized by species in Figure 2. The treatments are listed by category in Table 3. Full lists of the publications are available on the Open Science platform (https://doi.org/10.17605/OSF.IO/ST9MF accessed on 24 August 2023).

### 3.3. Risk of Bias

**Figure 3:**
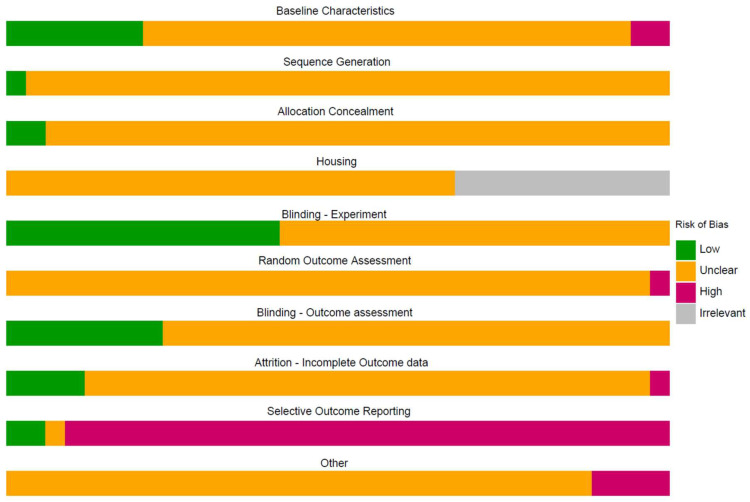
Summary of the risk of bias.

The median number of RoB items scored “unclear” per study was nine for animal and five for human treatment references, and the number ranged from three to nine. As in our preceding CF versus control analysis, human references scored significantly fewer “unclears,” reflecting more complete reporting than animal studies (W = 237.5, *p* < 0.001). The median number of RoB items scored “high” per references was one for both animal and human references. The median number of RoB items scored “low” per references was zero for animal and two for human references. The scores of the included treatment studies per item are shown in Figure 3, where the human references are listed in grey for the risk of bias related to housing.

### 3.4. Studies on Non-Viral Gene Therapy

The threshold to perform an MA, as specified in our protocol, was ≥3 animal model and ≥3 human studies reporting the nPD after treatment with a specific intervention. Based on the results shown in Table 1, which shows four NVGT studies in mice and four in humans, we were hopeful that overall, there would be enough data for an MA of the NVGT treatments. Additional data were thus extracted for the k = 8 NVGT references shown in Table 4. Full lists of the publications are available on the Open Science platform (https://doi.org/10.17605/OSF.IO/ST9MF accessed on 24 August 2023).

Publication dates for these references ranged from 1993 to 2002. Most of them described two-arm study designs comparing a treatment to a control. One study included both a sham and an untreated control [40], while another study described two distinct doses for NVGT [41]. All included NVGT references were written in English. Only three of the included references specifically mentioned the country of ethics evaluation, with two in the UK and one in the US. None of the eight included NVGT references mentioned a preregistration of the protocol, and seven referenced their methods.

Of the four human NVGT references, two described the inclusion of both genders, and two included males only. Sex was not mentioned in the four mouse references. The number of subjects studied ranged from 11 to 24 per reference. The nPD was tested from one to 18 times in these subjects. For our analyses, we selected a single nPD time point per included reference—the one closest to 24 h after treatment. For the included NVGT references, this resulted in nPDs from 16 h to 2 weeks after treatment, with substantial variation also within references. The NVGT treatment mainly (k = 7) comprised various kinds of liposomes, while only one study administered DNA complexes [42]. Control subjects were either untreated or received placebo/sham treatments.

After the control treatment, the baseline nPD ranged from 6.1 mV to 23.9 mV for mouse studies, and baseline nPD was 48.9 mV in the single included human study reporting this value. After NVGT treatment, the baseline nPD ranged from 13.1 mV to 23.9 mV for mouse studies and from 36.2 mV to 46.3 mV for human studies. Low chloride nPD values are not presented here because they were partially reported as a change to baseline and partially as absolute values.

**Figure 4:**
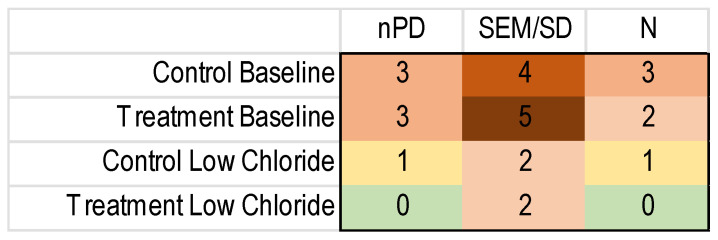
Heatmap of the missing nPD data in the included NVGT studies for the k = 8 NVGT treatment references. Green: no missing information. Yellow to dark red: increasing amounts of missing information.

To perform an MA, one needs the number of subjects and a measure of the variance besides the outcome measure for each included study. Unfortunately, the reporting of these important details was regularly lacking, as shown in Figure 4.

**Figure 5:**
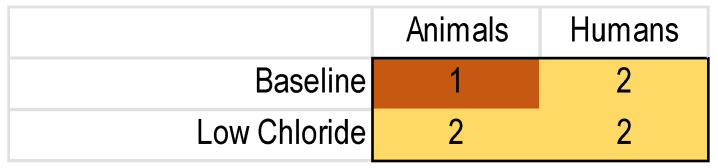
Heatmap of complete nPD data sets for NVGT treatments. Yellow and red reflect insufficient data; according to our protocol, we needed at least three of each population to perform a meta-analysis.

As the missing values were spread out over different studies, few of the data sets were complete (Figure 5). As this meant that the protocol-specified threshold was not met, we did not perform an MA.

## 4. Discussion

In this SR, we summarized the nPD after any CF treatment that was compared to a separate control group. We categorized the treatments into antibiotics [43], bone marrow transplant [44], CFTR protein [45], *CFTR* RNA [46,47], directly CFTR-targeting drugs [48,49], indirectly CFTR-targeting drugs (k = 11, refer to Table 1), NVGT (k = 8), VGT (k = 7), and other [50] treatments. As the first formal SR of the effects of CF treatments on the nPD, combining animal and human data, this could have been the leading data synthesis comparing efficacy between CF treatments, and treatment effects between animals and humans. However, there were large gaps in reporting, and the observed risk of bias was unclear for most included studies. Consequently, the overall evidence base per treatment category was too small to be conclusive, both for treatment effects and for animal–human comparability, even though we included more studies than many preceding reviews of animal studies, which has been reported to range, e.g., from 8 to 290 [23].

Even though our methods may look unfamiliar to those familiar with SRs of randomized clinical trials, the main strength this SR is its thorough methodology. The protocol was posted on the PROSPERO register [24] to prevent the cherry-picking of results, hypothesizing after the results were known (HARKing), and other teams duplicating our efforts [15]. We performed comprehensive searches in the two main medical databases, and all references were screened by two independent reviewers to minimize the chance of missing relevant records. To prevent errors, the study design parameters, outcome data, and risk of bias data were also extracted by two independent reviewers. To summarize, we followed all viable measures to minimize bias introduced during the review process.

The main limitation is the low amount of included data. There are three potential explanations: (1) incomplete sampling due to review methods; (2) the date of our search; or (3) lacking evidence within the published literature. Concerning sampling, the main limitation was that we only included between-subject comparisons and cross-over designs. Because within-subject comparisons can introduce bias due to time-associated factors, these were excluded. Based on the abstracts we read during screening, we estimated that including these less stringent experimental designs could have increased the amount of included data up to 30%, still leaving significant knowledge gaps.

Our search date of 23 March 2021 could be seen as a limitation. However, SRs generally take a long time to complete, with a median of 66 weeks and extremes up to 186 from start to completion [51]. With longer review durations, full review updates are crucial if the newly available literature could alter the conclusions of a review; thus, we tested if an update would be informative. To scope the amount of newly available literature, we repeated our searches in PubMed on 26 May 2023, with the publication date filtered to retrieve only references published after our original search. This search resulted in 87 hits. Rapid screening of these 87 hits was performed by a single reviewer (CL) in a single phase (exclude based on title and abstract or immediately retrieve PDF and include/exclude based on full text), with labelling for the treatment category during screening. Based on the title-abstract only, 75 references were excluded. Based on the full texts, another five references were excluded (three did not describe primary studies and two had the wrong study design). The full text of one reference [52] could not be retrieved directly. From the six included references, five were only relevant for our CF-Control comparisons [53,54,55,56,57]. The remaining reference, which would be included in a formal update of this part of our SR, described the effect of treatment with the CFTR-corrector c407 in mice [58]. Searching Embase in addition to PubMed, combined with further adaptations of the methods, may have resulted in a few additional references. However, based on this informal update, we do not expect a full update to change the conclusions of our review, and it would delay publication of these findings further.

In the opinion of the authors, further SRs of the nPD for CF treatmens are pointless until more primary data, resulting in a more informative overall evidence base, become available. In the meantime, preclinical SR efforts could focus on other important aspects of CF, such as the relationship between body weigh/body composition and clinical outcomes. Whereas several clinical reviews have assessed this subject (e.g., [59,60]), the preclinical evidence has not yet been reviewed. For overall CF treatment effects assessing multiple outcomes, the preclinical evidence base is also possibly relevant. Moreover, more meaningful analyses were possible in comparing baseline nPD values between animal models and patients, as shown in our other manuscript [16].

As described in the introduction, we defined an SR as a “review comprising a full search resulting in a complete literature overview, inclusion of papers following strict criteria, tabulation of extracted data, risk of bias assessment of included studies, and meaningful (qualitative or quantitative) synthesis of the data.” This definition is more stringent than some others (e.g., [27]), but we strongly encourage the use of other terms (mainly “mapping review” and “scoping review”) for reviews partially using systematic methods [15]. We followed our definition and synthesized the available data as meaningfully as possible, in line with previous SRs [27,61].

Whereas the nPD remains a reliable test for diagnosing CF and for testing treatment effects in animal models and human patients, there is a huge need to improve the reporting of the results to ensure reproducibility of the experiments within and between species. Particularly because the technique of measuring the nPD is complicated, and because different laboratories use different protocols, it is crucial for primary studies to report the actual outcome data together with the associated variance, numbers of subjects, and all study design parameters. This SR shows that missing data from several of the included studies made the planned analyses impossible, limiting the value of our efforts.

## Figures and Tables

**Table 1:** Full search strings.

PubMed	
nPD	Membrane potentials [MeSH:NoExp] OR ((Nasa*[tiab] OR naso*[tiab] OR membran*[tiab] OR transmembran*[tiab]) AND (Potential[tiab] OR potentials[tiab] OR voltage[tiab] OR voltages[tiab] OR current[tiab] OR currents[tiab]) AND (Difference[tiab] OR differences[tiab] or change[tiab] OR changes[tiab] OR alteration[tiab] OR alterations[tiab] OR variance[tiab]))
CF	Cystic fibrosis [MeSH] OR Mice, Inbred CFTR [MeSH] OR Cystic Fibrosis Transmembrane Conductance Regulator [MeSH] OR (cystic[tiab] AND (fibrosis[tiab] OR fibroses[tiab] OR fibrotic[tiab])) OR Mucoviscidos* [tiab] OR Mucoviscoid* [tiab] OR Mukoviszid* [tiab] OR CFTR [tiab] OR Fibrocystic Dis-ease [tiab] OR Fibrocystic Diseases [tiab] OR Mckusick [tiab] OR CFRD [tiab] OR “pancreas cystic disease” [tiab] OR muco-patient* [tiab] OR muko-patient* [tiab] OR (CF [tiab] AND (lung [tiab] OR lungs [tiab] OR pulmonary [tiab] OR ABPA [tiab] OR mucus [tiab] OR liver [tiab] OR livers [tiab] OR steatosis [tiab] OR cirrhosis [tiab] OR cirrhotic [tiab] OR meconium ileus[tiab] OR gastrointestinal [tiab] OR intestine [tiab] OR intestines [tiab] OR intestinal [tiab] OR duodenum [tiab] OR jejunum [tiab] OR colon [tiab] OR caecum [tiab] OR DIOS [tiab] OR ((sweat [tiab] OR eccrine [tiab] OR apocrine [tiab] OR salivary [tiab] OR parotid [tiab] OR sublingual [tiab] OR submandibular [tiab] OR sub-lingual [tiab] OR sub-mandibular [tiab] OR von Ebner [tiab]) AND (gland [tiab] OR glands [tiab])) OR ((Pa-ranasal [tiab] OR Para-nasal [tiab] OR frontal [tiab] OR ethmoidal [tiab] OR maxillary [tiab] OR sphenoidal [tiab]) AND (sinus [tiab] OR sinuses [tiab])) OR pancreas [tiab] OR pancreatic [tiab]))
Human	clinical study [pt] OR clinical trial [tiab] OR intervention study [tiab] OR “clinical studies as topic”[MeSH] OR first in man [tiab] OR proof of concept [tiab] OR randomized [tiab] OR placebo [tiab] OR drug therapy [sh] OR randomly [tiab] OR trial [tiab] OR groups [tiab] OR multicenter study[pt] OR “Multicenter Studies as Topic” [Mesh]
Animal	PubMed animal filter [25]
**Embase**	
nPD	exp potential difference/OR (exp nose/AND exp electrical parameters/) OR ((Nasa* OR naso* OR membran* or transmembran*) AND (Potential OR potentials OR voltage OR voltages OR current OR currents) AND (Difference OR differences or change OR changes OR alteration OR alterations OR variance)).ti,ab,kw.
CF	Cystic fibrosis/OR cystic fibrosis transmembrane conductance regulator/OR (cystic adj2 fibros*).ti,ab,kw. OR fibrocystic diseas*.ti,ab,kw. OR (mucovisc* or Mukoviszidose).ti,ab,kw. OR CFRD.ti,ab,kw. OR muco-patient*.ti,ab,kw. OR muko-patient*.ti,ab,kw. OR pancreas cystic disease.ti,ab,kw. OR pancreas fibrocystic disease.ti,ab,kw. OR pancreas fibrosis.ti,ab,kw. OR pancreatic cystic disease.ti,ab,kw. OR pancreatic fibrosis.ti,ab,kw. OR (CF adj30 (lung OR liver OR stomach OR intestines OR pulmonary OR meconeum ileus OR gastrointestinal OR intestine OR intestines OR intestinal OR pancreas OR pancreatic OR ((sweat OR eccrine OR apocrine OR salivary OR parotid OR sublingual OR submandibular OR von Ebner) adj2 (gland OR glands)) OR ((Paranasal OR frontal OR ethmoidal OR maxillary OR sphenoidal) adj2 (sinus OR sinusses)))).ti,ab,kw.
Human	exp clinical trial/OR clinical study/OR human subject.ti,ab,kw. OR clinical drug trial.ti,ab,kw. OR major clinical trial.ti,ab,kw. OR trial, clinical.ti,ab,kw. OR clinical study.ti,ab,kw. OR phase 1 clinical trial.ti,ab,kw. OR phase 2 clinical trial.ti,ab,kw. OR phase 3 clinical trial.ti,ab,kw. OR clinical trial, controlled.ti,ab,kw. OR clinical trial, phase 1.ti,ab,kw. OR clinical trial, phase 2.ti,ab,kw. OR clinical trial, phase 3.ti,ab,kw. OR clinical trials.ti,ab,kw. OR clinical trial, phase I.ti,ab,kw. OR clinical trial, phase II.ti,ab,kw. OR clinical trial, phase III.ti,ab,kw. OR intervention study.ti,ab,kw.
Animal	Embase animal filter [25]

**Table 2:** Exclusion criteria and the phases in which they were used.

	Screening Phase	
Exclusion Criterium ^1^	Title-Abstract	Full Text
Study not about CF	X	X
Study not in vivo ^2^	X	X
No nPD measured	X	X
No untreated control group (either with or without CF) ^3^ present		X
No full peer-reviewed publication ^4^		X

^1^ There were no restrictions for publication date or language. ^2^ Ex vivo, in vitro and in silico models were excluded. ^3^ For the full review, we included both studies that compared CF with healthy controls at baseline and studies that compared treated with untreated CF. In this publication we only present the second category, as selected in the first phase of data extraction (see below). ^4^ Conference proceedings and short communications lacking a detailed description of the methods were excluded because we planned analyses of the experimental set-ups.

**Table 3:** Treatments for which nPD data were published.

Study ID ^1^	Title	Population	Treatment
**Antibiotics**			
Barker_2005	Effect of macrolides on in vivo ion transport across cystic fibrosis nasal epithelium	Humans	Clarithromycin; Azithromycin
**Bone Marrow Transplant**			
Bruscia_2006	Assessment of cystic fibrosis transmembrane conductance regulator (CFTR) activity in *CFTR*-null mice after bone marrow transplantation	Animals	
**Direct CFTR targeting drugs**			
Accurso_2014	Sweat chloride as a biomarker of CFTR activity: proof of concept and ivacaftor clinical trial data	Humans	Ivacaftor
Rowe_2013	Optimizing nasal potential difference analysis for CFTR modulator development: assessment of ivacaftor in CF subjects with the G551D-*CFTR* mutation	Humans	Ivacaftor
**Indirect CFTR-affecting drugs**			
Cartiera_2010	Partial correction of cystic fibrosis defects with PLGA nanoparticles encapsulating curcumin	Animals	Curcumin
Clancy_2012	Results of a phase IIa study of VX-809, an investigational CFTR corrector compound, in subjects with cystic fibrosis homozygous for the F508del-*CFTR* mutation	Humans	VX-809
Egan_2002	Calcium-pump inhibitors induce functional surface expression of DELTAF508-CFTR protein in cystic fibrosis epithelial cells	Animals	Thapsigargin
Egan_2004	Curcumin, a major constituent of turmeric, corrects cystic fibrosis defects	Animals	Curcumin
Kerem_2014	Ataluren for the treatment of nonsense-mutation cystic fibrosis: a randomised, double-blind, placebo-controlled phase 3 trial	Humans	Ataluren
Lubamba_2008	Preclinical evidence that sildenafil and vardenafil activate chloride transport in cystic fibrosis	Animals	Sildenafil and Vardenafil
Lubamba_2009	Airway delivery of low-dose miglustat normalizes nasal potential difference in F508del cystic fibrosis mice	Animals	Miglustat
Lubamba_2011	Inhaled phosphodiesterase type 5 inhibitors restore chloride transport in cystic fibrosis mice	Animals	Type-5 phosphodiesterase inhibitors
McCarty_2002	A phase I randomized, multicenter trial of CPX in adult subjects with mild cystic fibrosis	Humans	CPX
Rubenstein_1998	A pilot clinical trial of oral sodium 4-phenylbutyrate (Buphenyl) in deltaF508-homozygous cystic fibrosis patients: partial restoration of nasal epithelial CFTR function	Humans	Buphenyl (sodium-4-phenylbutyrate)
Zeitlin_2002	Evidence of CFTR function in cystic fibrosis after systemic administration of 4-phenylbutyrate	Humans	Buphenyl (sodium-4-phenylbutyrate)
**Non-Viral Gene Transfer (NVGT)**			
Alton_1993	Non-invasive liposome-mediated gene delivery can correct the ion transport defect in cystic fibrosis mutant mice	Animals	*CFTR* cDNA-liposome complexes (*DC-Chol*/*DOPE*)
Alton_1999	Cationic lipid-mediated *CFTR* gene transfer to the lungs and nose of patients with cystic fibrosis: a double-blind placebo-controlled trial	Humans	Cationic lipid-mediated gene transfer (GL-67/DOPE/DMPE-PEG500)
Caplen_1995	Liposome-mediated *CFTR* gene transfer to the nasal epithelium of patients with cystic fibrosis	Humans	Complementary DNA in liposomes
Gill_1997	A placebo-controlled study of liposome-mediated gene transfer to the nasal epithelium of patients with cystic fibrosis	Humans	Liposome-mediated gene transfer
Hyde_2000	Repeat administration of DNA/liposomes to the nasal epithelium of patients with cystic fibrosis	Humans	DNA/liposomes
Jiang_1998	Efficiency of cationic lipid-mediated transfection of polarized and differentiated airway epithelial cells in vitro and in vivo	Animals	CL-67 mediated gene transduction (cationic lipid)
McLachlan_1996	Laboratory and clinical studies in support of cystic fibrosis gene therapy using *pCMV-CFTR-DOTAP*	Both	Gene therapy using *pCMV-CFTR-DOTAP*
Ziady_2002	Functional evidence of *CFTR* gene transfer in nasal epithelium of cystic fibrosis mice in vivo following luminal application of DNA complexes targeted to the serpin-enzyme complex receptor	Animals	*CFTR* gene transfer with serpin-targeted molecular conjugates
**Other**			
Lazrak_2014	Inter-α-inhibitor blocks epithelial sodium channel activation and decreases nasal potential differences in ΔF508 mice	Animals	Inter-alpha inhibitor
**Protein**			
Ramjeesingh_1998	Assessment of the efficacy of in vivo CFTR protein replacement therapy in CF mice	Animals	Purified CFTR protein via phospholipid liposomes
**RNA**			
Beumer_2019	Evaluation of eluforsen, a novel RNA oligonucleotide for restoration of CFTR function in in vitro and murine models of p.Phe508del cystic fibrosis	Animals	Eluforsen
Robinson_2018	Lipid Nanoparticle-Delivered Chemically Modified mRNA Restores Chloride Secretion in Cystic Fibrosis	Animals	lipid nanoparticle-delivered chemically modified mRNA
**Viral Gene Transfer (VGT)**			
Cmielewski_2014	Long-term therapeutic and reporter gene expression in lentiviral vector treated cystic fibrosis mice	Animals	*CFTR* gene in lentiviral vector
Grubb_1994	Inefficient gene transfer by adenovirus vector to cystic fibrosis airway epithelia of mice and humans	Both	Gene transfer by adenovirus
Jiang_1997	Increased contact time improves adenovirus-mediated *CFTR* gene transfer to nasal epithelium of CF mice	Animals	Adenovirus-mediated gene transfer
Limberis_2002	Recovery of airway cystic fibrosis transmembrane conductance regulator function in mice with cystic fibrosis after single-dose lentivirus-mediated gene transfer	Animals	Lentivirus-Mediated Gene Transfer
Ostedgaard_2002	*CFTR* with a partially deleted R domain corrects the cystic fibrosis chloride transport defect in human airway epithelia in vitro and in mouse nasal mucosa in vivo	Animals	Adenoviral vectors
Parsons_1998	Enhanced in vivo airway gene transfer via transient modification of host barrier properties with a surface-active agent	Animals	Adenoviral gene transfer
Vidovic_2016	rAAV-*CFTR*Δ*R* Rescues the Cystic Fibrosis Phenotype in Human Intestinal Organoids and Cystic Fibrosis Mice	Animals	rAAV vector containing truncated *CFTR*

^1^ Full citations are available in the data files on the open science platform.

**Table 4:** NVGT studies.

Study_ID ^1^	Ethics Review	Species	Sex/Gender	N Studied	Number of Repetitions	Time after Treatment
Alton_1993		Mice	nr	16	1	16–68 h
Alton_1999	UK	Humans	Males	16	8	1 day
Caplen_1995		Humans	Males	15	6	1 day
Gill_1997A		Humans	Both	12	11	mean value of day 10, 12 and 14
Gill_1997B		Humans	Both	12	11	mean value of day 10, 12 and 14
Hyde_2000	UK	Humans	Both	12	18	Days 2–7
Jiang_1998		Mice	nr	15	1	nr

^1^ Full citations are available in the data files on the open science platform.

## Data Availability

All used search strings are publicly available within one of the references. All search results, screening results, and extracted data are available on OSF (https://doi.org/10.17605/OSF.IO/ST9MF).
